# The Diagnosis of Human Fascioliasis by Enzyme-Linked Immunosorbent Assay (ELISA) Using Recombinant Cathepsin L Protease

**DOI:** 10.1371/journal.pntd.0002414

**Published:** 2013-09-19

**Authors:** Bibiana Gonzales Santana, John P. Dalton, Fabio Vasquez Camargo, Michael Parkinson, Momar Ndao

**Affiliations:** 1 National Reference Center for Parasitology, the Research Institute of the McGill University Health Center, Montreal, Quebec, Canada; 2 Institute of Parasitology, McGill University, Ste-Anne-de-Bellevue, Quebec, Canada; 3 School of Biotechnology, Dublin City University, Glasnevin, Dublin, Ireland; Centers for Disease Control and Prevention, United States of America

## Abstract

**Background:**

Fascioliasis is a worldwide parasitic disease of domestic animals caused by helminths of the genus Fasciola. In many parts of the world, particularly in poor rural areas where animal disease is endemic, the parasite also infects humans. Adult parasites reside in the bile ducts of the host and therefore diagnosis of human fascioliasis is usually achieved by coprological examinations that search for parasite eggs that are carried into the intestine with the bile juices. However, these methods are insensitive due to the fact that eggs are released sporadically and may be missed in low-level infections, and fasciola eggs may be misclassified as other parasites, leading to problems with specificity. Furthermore, acute clinical symptoms as a result of parasites migrating to the bile ducts appear before the parasite matures and begins egg laying. A human immune response to Fasciola antigens occurs early in infection. Therefore, an immunological method such as ELISA may be a more reliable, easy and cheap means to diagnose human fascioliasis than coprological analysis.

**Methodology/Principal findings:**

Using a panel of serum from *Fasciola hepatica*-infected patients and from uninfected controls we have optimized an enzyme-linked immunosorbent assay (ELISA) which employs a recombinant form of the major *F. hepatica* cathepsin L1 as the antigen for the diagnosis of human fascioliasis. We examined the ability of the ELISA test to discern fascioliasis from various other helminth and non-helminth parasitic diseases.

**Conclusions/Significance:**

A sensitive and specific fascioliasis ELISA test has been developed. This test is rapid and easy to use and can discriminate fasciola-infected individuals from patients harbouring other parasites with at least 99.9% sensitivity and 99.9% specificity. This test will be a useful standardized method not only for testing individual samples but also in mass screening programs to assess the extent of human fascioliasis in regions where this zoonosis is suspected.

## Author Summary

Fascioliasis is a food-borne human disease caused by helminth parasites of the genus Fasciola. It is a global disease of domestic animals but its increased recognition as a major zoonosis has led to the World Health Organization including fascioliasis on the list of important human parasitic diseases. Current diagnosis of human fascioliasis involves the detection of eggs in the stool. However, eggs are not observed during the acute phase when the parasite is migrating through the tissues, and can be missed during the chronic phase when parasites are in the bile duct due to the sporadic release of the bile into the intestines. Using a panel of serum from *Fasciola hepatica*-infected patients, we have optimized an enzyme-linked immunosorbent assay (ELISA) which employs a recombinant form of the major *F. hepatica* cathepsin L1 as the antigen for the diagnosis of human fascioliasis. The test is easy to use and can discriminate fasciola-infected individuals from patients harbouring other parasites with 99.9% sensitivity and 99.9% specificity. This ELISA will be a useful standardized method not only for testing individual samples but also in mass screening programs to assess the extent of human fascioliasis in regions where this zoonosis is suspected.

## Introduction

Fascioliasis, or liver fluke disease, is a food-borne infection caused by trematodes of the genus Fasciola. The disease has been traditionally viewed as of mainly veterinary importance because of the substantial production and economic losses it causes in livestock, particularly sheep and cattle. In contrast, human fascioliasis has until recently been neglected by the medical community. Due to its increased spread and chronic nature, it is now recognized as a disease of global human concern by the (WHO) [1]–[3]. Studies indicate that approximately 17 million people are infected with Fasciola and 91.1 million are living at risk of infection [4].

*Fasciola hepatica* has a worldwide distribution and causes major health problems in Europe (Portugal, France and Spain), the Americas (Bolivia, Peru, Chile, Ecuador and Venezuela), Cuba and Oceania and overlaps with *F. gigantica* in many areas of Africa and Asia [5]. Interestingly, high prevalence in humans does not appear to be related to high prevalence in livestock, so that the expected correlation between animal and human fascioliasis is not a consistent finding [6]. On the other hand, *Fasciola* g*i*g*antica*, in humans was thought to be of relatively little importance due to its low incidence in endemic areas. However, since fascioliasis is not a reportable disease in many countries, the number of cases (>500) reported in the literature represent the tip of the iceberg [7], [8].

*F. hepatica* tolerates a wide range of environmental conditions and has a remarkable ability to adapt to new hosts [9] and thus has a wide host range [5]. This has led to its spread from its original location in pre-domestication of animals and more recently over the five continents due to the export of European livestock during colonization [10]. The spread of *F. hepatica* is also related to the geographic expansion of its original intermediate host, the snail G*alba truncatula*. By contrast, the smaller geographic distribution of *F.* g*i*g*antica* seems to be related to the weaker diffusion capacity of its intermediate snail hosts (African *Radix natalensis* and the Eurasian *Radix auricularia)* [6]. The most commonly affected are farm animals (eg, sheep and cattle). Nevertheless, it can infect a variety of wild animals (eg, deer, llamas, kangaroos, rabbits, beavers, and rats) which shows the remarkable capability of the parasite to adapt to new hosts [6], [9].

Infections in animals and humans occur when vegetation or water contaminated with infective encysted dormant larvae (metacercariae) is ingested. The parasites excyst in the host intestine, migrate through the intestinal wall into the peritoneal cavity and then into the liver parenchyma where they caused extensive tissue damage and blood vessel hemorrhaging representing the acute phase of the disease [11]. After eight to twelve weeks post infection the parasites move into the biliary passages, become sexually mature and start producing eggs [9]. The parasites become obligate blood feeders on host haemoglobin to support the production of eggs and access the blood by puncturing the bile ducts wall [12]. *Fasciola spp.* have been estimated to produce 20 000 to 50 000 eggs per fluke per day in animals [13].

Up to 50% of *F. hepatica* infections are asymptomatic and disease may appear anywhere from a few days to several years after infection thereby making the diagnosis challenging [14]. Human fascioliasis is routinely diagnosed by the detection of parasite eggs in the feces. These can only be detected after the parasite has matured in the bile ducts and released eggs that are carried into the intestine with the bile juices. However, this coprological method presents several drawbacks: First, bile juices are irregularly released into the intestine and therefore more than one samples needs to be assessed. Second, in low level infections the fluke eggs are often not found in the feces, even after multiple fecal examinations [15]. Third, since eggs are produced by mature adults in the bile ducts, the acute phase of the disease is are not identified.

Enzyme-linked immunosorbent assay (ELISA) methods developed for determination of anti-*Fasciola* antibodies provide an alternative to coprological examination. Anti-*F. hepatica* antibodies can be found after 2–4 weeks post-infection providing a means for early detection of disease using parasite extracts or excretory-secretory (FhES) products as antigen [16]. Cathepsin Ls proteases (FhCL), the most predominant component of FhES have been employed for the development of enzyme linked immunosorbant assays (ELISA) and proven to be highly effective in a number of diagnostic studies performed in our laboratory in the past few years [17]–[19]. FhCL1 was initially purified from ES antigens by Smith and others [20] and shown to be released by vesicles synthesized by the intestinal cells of the liver fluke [21] indicating a role in the digestion of ingested blood and tissues. The protease is also likely to have a role in assisting the parasites' migration through the host's tissues [22] since it is capable of degrading the extracellular matrix and basal membrane components and may also have a role in evasion of immune response since it can cleave host immunoglobulins and prevent attachment of immune effector cells to newly excysted juveniles (infective larvae) [20], [23].

Previous studies used native FhCL1 or an enzymatically active recombinant rFhCL1. However these were prone to breakdown and auto-catalytic degradation during purification and also cleaved immunoglobulin in the ELISA. We have therefore employed an inactive recombinant FhCL1 variant (FhCL1Gly^26^). We also employed more recently developed and commercially available secondary antibodies against anti-total IgG and several subclasses (IgG1, IgG2 and IgG4) to screen and optimize the test using a panel of serum samples well-characterized Fasciola-infected patients. The test we developed is easy to use and can discriminate fasciola-infected individuals from patients harbouring other parasites with 99.9% sensitivity and 99.9% specificity. This ELISA will be a useful standardized method not only for testing individual samples but can be employed in mass screening programs to assess the extent of human fascioliasis in regions where this zoonosis is suspected.

## Materials and Methods

### Reagents

High protein-binding 96-well polystyrene microtiter plates were purchased from Thermo Fischer Scientific Inc. (Cat #3455, Ontario Canada), Peroxidase-conjugated labeled anti-human immunoglobulin IgG (Goat) was from Perkin Elmer (Cat #NEF802, Massachusetts, USA). The substrate 3,3′,5,5′-Tetramethylbenzidine (TMB/E) was purchased from Millipore (Cat # ES001-500 ml, Massachusetts, USA). Peroxidase-conjugated anti-human immunoglobulin IgG1, IgG2 and IgG4 were purchased from Southern Biotech (Cat # 9052-5, 9060-05, 9190-05 respectively, Birmingham, Alabama, USA).

### Clinical samples

The human Fasciola samples were reviewed and approved by the ‘Pedro Kourí’ Tropical Medicine Institute (IPK, Havana City, Cuba) Biomedical Research Ethics Board. The human control and other parasitic diseases sera were obtained from the Passive Parasitic Diseases Surveillance System diagnostic testing at the National Reference Laboratory for Parasitology (NRCP; Montreal, Quebec, Canada) and were considered exempt. All samples used in this study were anonymized. These consisted of samples from 93 Cuban individuals that were coprologically-positive for eggs of *F. hepatica* and clinically diagnosed in the hospital, samples from 72 Cuban and 63 Canadian individuals that were shown to be negative for Fasciola infection, and 158 serum samples obtained from individuals infected with other parasitic diseases including, amoebiasis (12), ascariasis (10), Chagas disease (10), cysticercosis (10), echinococosis (13), enterobiasis (2), filariasis (11), giardiasis (5), leishmaniasis (9), malaria (14), metorchiasis (9), schistosomiasis (9) , strongyloidiasis (6), toxocariasis (14), toxoplasmosis (13), and trichinellosis (11).

### Expression and purification of FhCL1

The full length *F. hepatica* preprocathepsin L1 cDNA was previously cloned in our laboratory into a *P. pastoris* multicopy system using P. *pastoris* GS115 strain and pPIC9K vector *^(26)^*. The variant FheproCL1Gly^26^ (Cys^26^ to Gly^26^) was used in this study and expressed as described by Collins et al. *^(21)^*. The inactive enzyme was produced by fermentation at 30°C and 250 rpm in 1 liter BMGY broth buffered to pH 6.0 into 4 liter baffled flasks until an OD_600_ of 2–6 was achieved. The cells were centrifuged at 3,000× g for 10 minutes at room temperature and the induction initiated by resuspending the pellets in 200 ml BMMY broth and adding 1% of filter–sterilized 100% methanol every 24 hours for 3 days. The culture was then centrifuged at 16,000× g for 30 minutes at room temperature. The pellets were discarded and FhCL1 isolated from the supernatant by Ni-NTA affinity chromatography as previously described [17], [18].

### Optimization and development of the enzyme-linked immunosorbent assay (ELISA)

For the purpose of optimizing the ELISA a pool of serum from fasciola-infected individuals (30) and of negatives controls (30) was prepared. Determination of the optimum antigen concentration and the dilution of the sample serum and secondary conjugated antibody that gave the most superior background-to-signal ratio were assessed by employing a matrix formation. Using different 96-well plates, each with a constant antigen concentration, different dilutions of the pooled positive control serum was added to the wells from top to bottom (well A–G) while different dilutions of the secondary antibody were tested in duplicate from left to right (wells 1–12). All optimization experiments were repeated at least once.

For each plate FhCL1 antigen was dissolved in bicarbonate/carbonate coating buffer at pH 9.0. One hundred microliters of the solution was then added to each well and incubated overnight at 4°C. After washing four times, excess protein binding sites were blocked at 37°C for 1 h by adding 100 µl of 2% bovine serum albumin diluted in PBS/0.1% Tween 20. After a further washing procedure, 100 µl of pooled samples sera (diluted at 1∶100, 1∶200, 1∶400 and 1∶800) were added and the plate incubated for 1 h at 37°C. Following another wash, 100 µl of peroxidase-conjugated anti-human IgG (diluted 1∶4000, 1∶8000, 1∶12000, 1∶16000 and 1∶32000) was added to the wells and the plates were incubated for 30 min at 37°C. After a final washing step bound antibodies were detected by the addition of 100 µl of TMB. The color was developed for 10 min and the reaction was stopped with 50 µl of 0.1 M sulphuric acid. The plates were read on an ELISA plate reader at 405 nm. All serum samples were analyzed for the binding of total IgG and IgG1, IgG2 or IgG4 using the appropriate secondary monoclonal antibodies specific for each. Results are reported as the mean values obtained from three independent experiments conducted in duplicate.

### Statistical analysis

Box-Cox transformation of the data from uninfected control for IgG showed that lambda of 0.33 minimized skewness in the data. Data was therefore transformed by cube-rooting to normalize the distribution prior to statistical analysis [24]. A standard deviation for the uninfected controls and for the fascioliasis positive was determined from the transformed data and a cut-off limit for sensitivity and specificity in the assay set at t-standard deviations from the mean for a one-tailed test with p = 0.0001. This was converted back to the original units by cubing. Homogeneity of variance was assessed by Levene's test. Effect of infection on IgG absorbance was assessed by Oneway ANOVA. The difference between infected and control was assessed by post-hoc testing with Dunnett's test for each disease against the control, with a one-sided test. All statistical analysis was carried out using SPSS version 17. Differences between negative peaks were analyzed by the Mann-Whitney U-Test. Normality was assessed by the Shapiro-Wilk test.

## Results

### Optimization of ELISA using cathepsin L1 for diagnosis of Fascioliasis

To determine the optimal conditions for diagnosis of human fascioliasis by ELISA using the FhCL1 as antigen we used a pool of positive control sera from 20 Cuban patients with a known infection with *F. hepatica* and a pool of sera from 20 Cuban patients negative for this parasitic infection. We performed a matrix comparison of ELISAs using various antigen concentrations, dilutions of the pooled primary sera and dilutions of secondary antibodies specific for different human antibody isotypes. The ELISA conditions providing the best positive to negative signal ratio and used in our subsequent studies were as follows: wells were coated with 100 µl of 0.25 µg/ml of the FhCL1 antigen; the dilution of the human primary sera used was 1/200 and the dilution of the secondary antibody was 1/32000, 1/8000, 1/100 and 1/32000 for secondary antibodies anti-total IgG, anti-IgG1, anti-IgG2 and anti-IgG4, respectively.

### Validation of ELISA using FhCL1 for diagnosis of Fascioliasis

**Figure 1:**
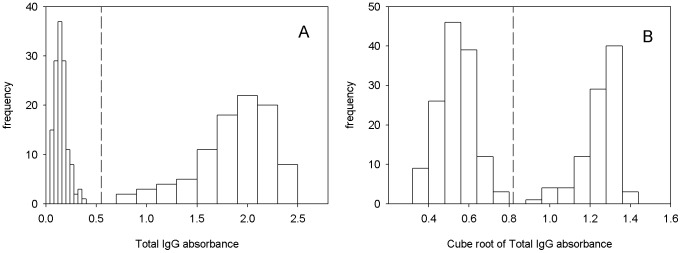
Analysis of ELISA results using FhCL1 antigen and anti-total-IgG as secondary antibody. A) Histogram of control and *Fasciola-*positive serum samples. B) Histogram of normalized total IgG for negative control and *Fasciola* positive samples. Dash line represents cut-off for negative samples.

A total of 386 serum samples were screened using our optimized ELISA. Statistical analysis of the ELISA data was performed to evaluate the efficacy of FhCL1 to discriminate between positive infected individuals and negative non-infected individuals. First, the results for assays using anti-total IgG as the secondary antibody were plotted in a histogram to evaluate the distribution of the population to be analyzed (Figure 1A). The data were normalized for statistical analysis by cube rooting (Figure 1B). Using the normalized data, the standard deviations for the negative and positive peaks were calculated to establish the cut-off limit for the sensitivity and specificity of the assay to detect non-infected and infected individuals. The cut-off for the negatives for the transformed data was therefore set at 0.82 OD units with p = 0.0001 using a one-tailed test which separates 99.99% of the uninfected patients to the left of the line and infected patients to the right (black vertical dashed line in the histogram (Figure 1B). This value was then converted back into the normal data and gave a cut-off of 0.55 OD units (Figure 1A). The cut-off for the infected positive patients was computed in the same manner as the negative patients and gave a value of 0.58 OD units for the normal data (data not shown in histogram). It can be observed in Figure 1A that no Fasciola-negative patients fell to the right of the cut-off, and no Fasciola-positives fell to the left. Therefore, not only did the ELISA test using anti-total IgG secondary antibody give a 99.99% specificity but it also exhibited a >99.99% sensitivity for identifying infected individuals.

**Figure 2:**
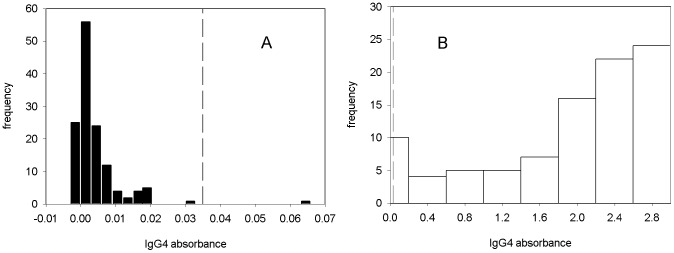
Sensitivity and specificity analysis for ELISA using anti-IgG4 as secondary antibody. A) Histogram showing negative sample where two fall out of the cut-off limit. B) Histogram showing Fasciola-positive and control negative sera. Discrimination between negatives and positives exists; however, a small space exists between them that cannot be noticed from the graph. Dashed line represents the cut-off for negatives sample.

Statistical analysis of the data obtained using anti-IgG4 as the secondary antibody was also performed. The data was plotted into a histogram and results analyzed. Two of the negatives samples do not seem to belong to the distribution of the rest of the negatives and are outside the cut-off that discriminate 99.99% of the uninfected patients (Figure 2A). To group the entire Fasciola-negative individuals together the cut-off limit was set just below the positives at 0.1 OD units. The cut-off was set then at 4.2 OD units standard deviations from the mean of the negative patients giving a cut-off of 0.1 OD units (p = 0.0001) that provided a 99.99% discrimination between positives and negatives. When we plotted the positives and negatives patients on a histogram a large spread of positives was observed and only a spike for negatives was found (the values for negatives are very low) (Figure 2B). However, while we found that using anti-IgG4 secondary antibodies had the potential to discriminate between positives and negative infected patients, the gap between these was very small (this cannot be fully appreciated in the graph shown in Figure 2B as the bars divided by the dashed cut-off line lie next to each other) and therefore more probability of error.

**Figure 3:**
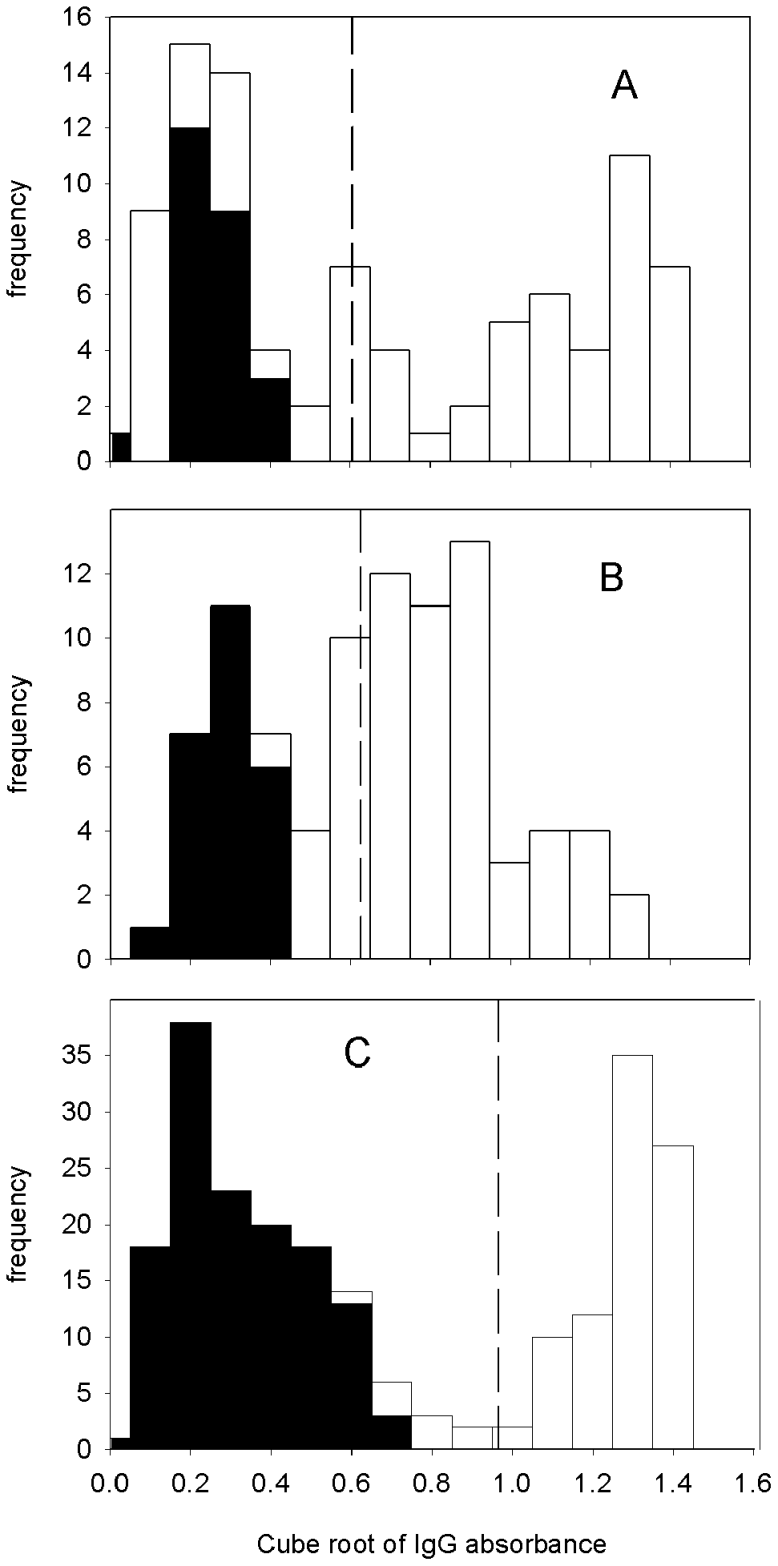
Analysis of ELISA data using FhCL1 antigen and secondary antibodies specific for IgG1, IgG2 and IgG4+IgG1 isotypes. Histograms of ELISA data obtained for Fasciola-positive and control patient sera using A) anti-IgG1, B) anti-IgG2, and C) IgG4+IgG1 secondary antibodies. Black bars represent sera from control patients while white bars represent sera from positive patients.

When we employed secondary antibodies specific for IgG1 and IgG2 in our ELISA assays the sensitivity and specificity dropped drastically compared to anti-total IgG and anti-IgG4. For these assays we found that the data was badly skewed from a normal distribution and a clear cut-off between the negative and positive patient sera could not be established. Thus a definitive distinction of non-infected and infected patients could not be made (Figure 3A, 3B and 3C).

### Comparison of isotypes in FhCL1 ELISAs using scattergraphs

**Figure 4:**
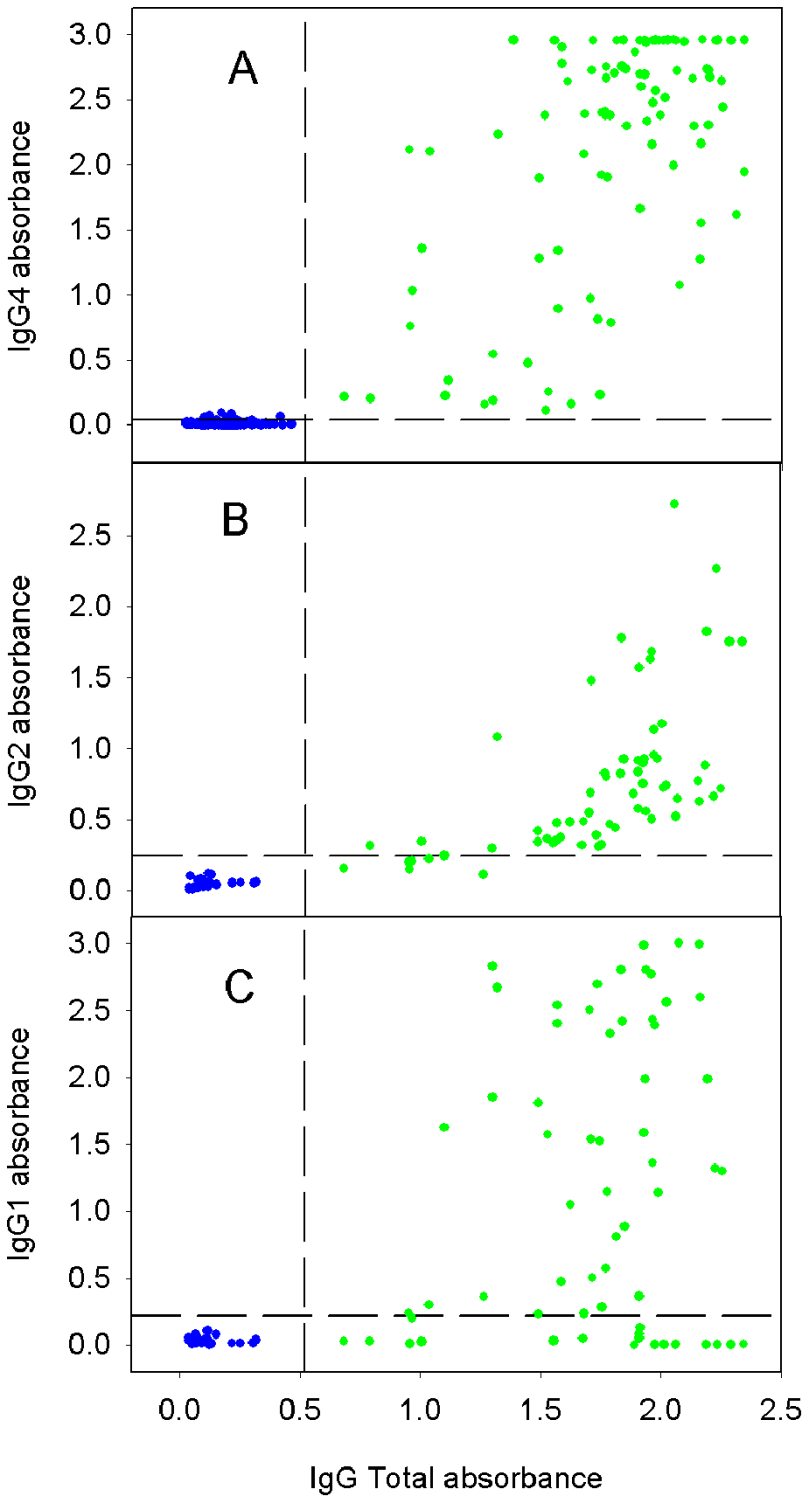
Scattergraphs for ELISA data using various secondary antibodies. Data obtained using secondary antibodies specific for anti-IgG4 (A), anti-IgG2 (B) and anti-IgG1 (C) compared to data obtained using anti-total IgG. Blue circles represent sera from control patients while green circles represent Fasciola-positive patients.

To visualize the difference between the results obtained for the various specific isotypes more clearly, we compared the data using scattergraphs. It can be seen in Figure 4A that for IgG4 some space separated the *F. hepatica* negative and positive patients although the gap was small, as found with the histogram. Both anti-IgG1 and anti-IgG2 were even less effective with a large overlap between the Fasciola-negatives giving the highest readings and the Fasciola-positives giving the lowest readings (Figure 4B and C). Using these latter two secondary antibodies it seems inevitable that we could get many of false positives and negatives.

**Figure 5:**
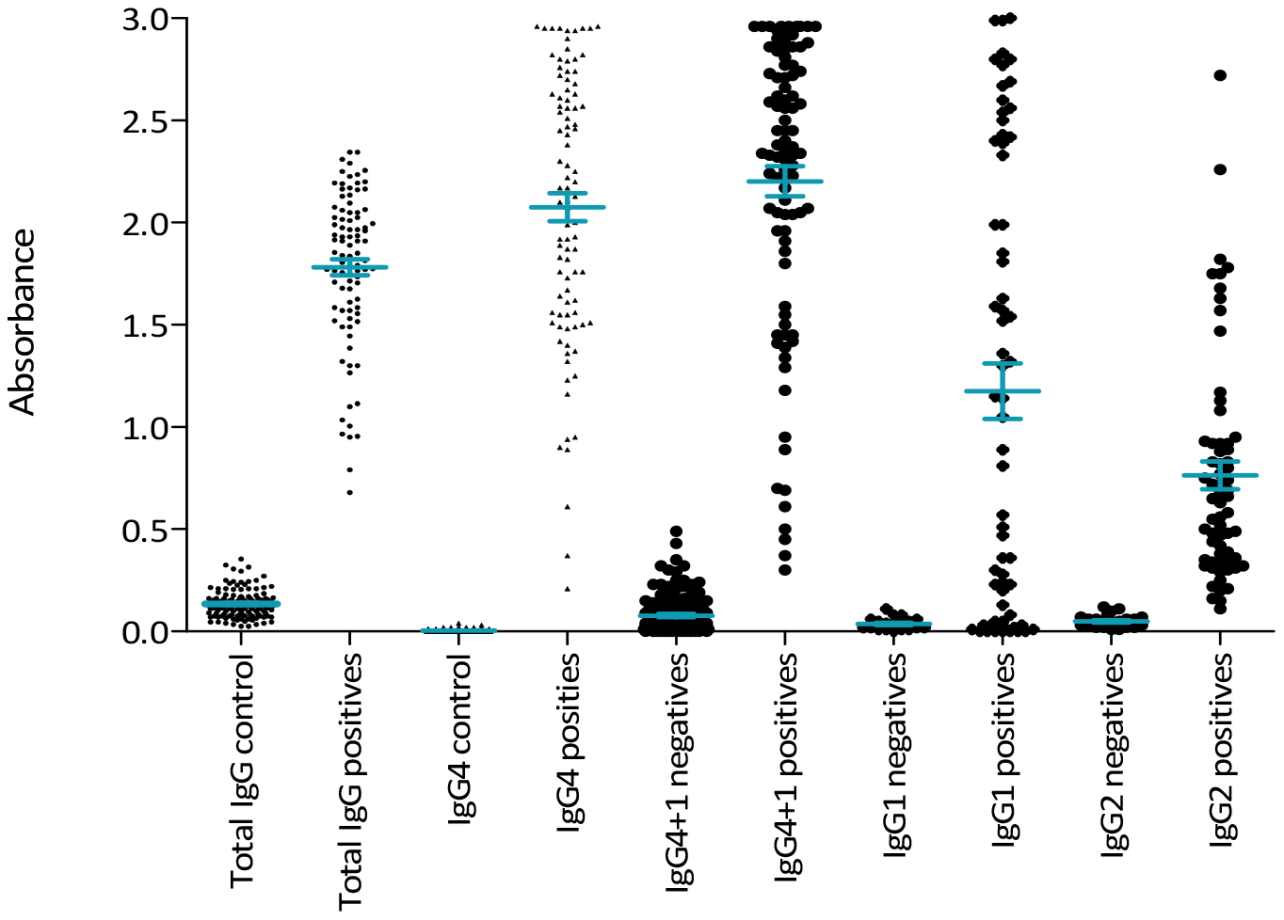
Comparison of ELISA data obtained using secondary antibodies specific for various isotypes. Absorbance between positive infected sera and negative control sera using anti-total IgG and the different serotypes.

Figure 5 shows a comparison between the data using anti-total IgG secondary antibody with the other secondary antibodies and summarizes the results. The results from fasciola-infected (positive) and non-infected (control) sera for each secondary antibody were plotted separately. It is clear that using anti-total IgG provides best discrimination between positives and negative samples. While the difference between the mean values for the positive and negative samples was wider when anti-IgG4 was used in the assays, the spread of readings obtained for the positive samples reduced the ability to distinguish between the borderline cases and the negative patients compare to anti-total IgG. The overlap of positives and negatives was even more pronounced when anti-IgG1 and IgG2 alone were applied.

### Analysis of sera from various parasitic infections in FhCL1 ELISAs

**Figure 6:**
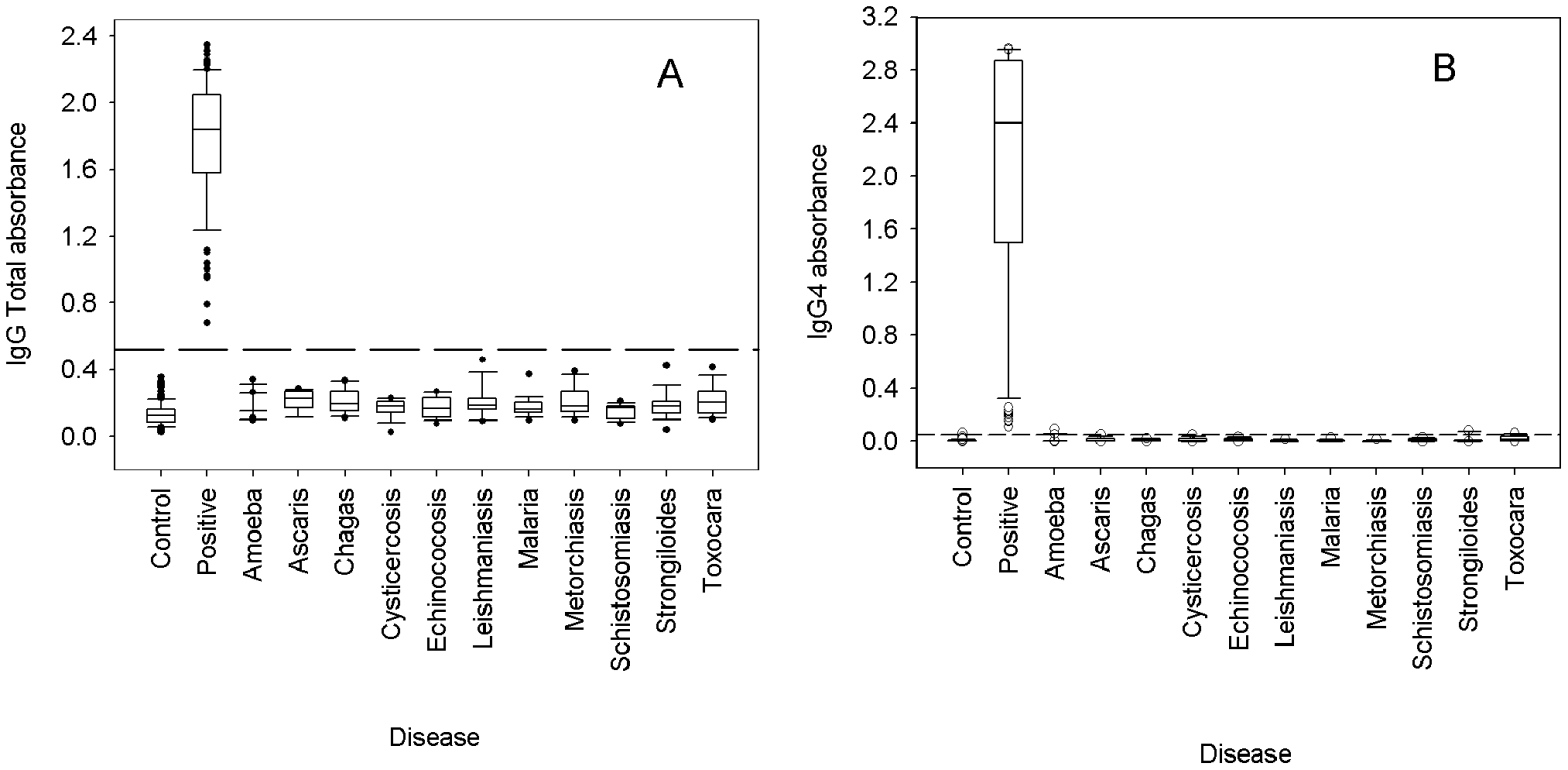
Box plots of ELISA data using sera from non-infected control patients, *F. hepatica*-infected patients, and patients with various parasitic diseases. A: ELISA using anti-total IgG as secondary antibody and B: ELISA using anti-IgG4 as secondary antibody. The dashed line represents the cut-off for negative samples. Results are obtained from three independent experiments conducted in duplicate.

To examine if cross-reactivity of our ELISA using recombinant FhCL1 for the detection of human fascioliasis was evident, we performed assays using fasciola-infected patient sera (93 infected Cubans) and non-infected (72 Cubans and 63 Canadians) and compared these with sera obtained from patients infected with range of other worm as described above. We used anti-total IgG and anti-IgG4 as the secondary antibodies (Figure 6).

The result showed that absorbance readings obtained with sera from patients infected with parasites other than *F. hepatica* closely matched that obtained with the negative control samples. We found that using 0.55 OD units as cut-off with anti-total IgG as secondary antibody, the test can discriminate between *F. hepatica* patients and all other infections examined (Figure 6A). Using Oneway ANOVA we found a very highly significant effect of treatment (disease) (p<0.0005), and post-hoc comparison of all parasitic diseases compared to the negative controls using Dunnet's test showed that none of these had a significantly greater IgG value than the controls except for fascioliasis using one-tailed test at 0.01% level. These results revealed two homogeneous populations within the data and suggest a very high level of confidence for distinguishing between *F. hepatica* and the other diseases tested. Thus, no evidence of cross-reactivity was observed (table 1). A similar analysis was computed with the data obtained with anti-IgG4 using a 0.1A OD units cut-off line. *Fasciola* positives can be differentiated from controls and all other infections, although the discrimination between these was smaller when compared with the anti-total IgG data (Figure 6B).

**Table 1:** Absorbance values of total IgG.

Group	N of individuals	Mean	Std. Deviation
Control	135	0.134	0.065
Positives	93	1.780	0.374
Amoebiasis	12	0.200	0.054
Ascariasis	10	0.214	0.063
Chagas disease	10	0.210	0.076
Cysticercosis	10	0.170	0.059
Echinoccocosis	13	0.174	0.067
Enterobiasis	2	0.170	0.091
Filariasis	11	0.086	0.033
Giardiasis	5	0.199	0.037
Leishmaniasis	9	0.211	0.109
Malaria	14	0.179	0.066
Metorchiasis	9	0.216	0.096
Schistosomiais	9	0.147	0.045
Strongyloidiasis	6	0.184	0.057
Toxocariasis	14	0.191	0.089
Toxoplasmosis	13	0.214	0.096
Trichinellosis	11	0.085	0.052

Results are obtained from three independent experiments conducted in duplicate.

## Discussion

Previous studies in our laboratory have shown the potential of *F.hepatica* cathepsin L1 antigen to detect Fascioliasis with high confidence [17]–[19], [25]. However, in these studies we employed either a native form of cathepsin L1 isolated from the secretory products of the parasite or an enzymatically active recombinant cathepsin L1 expressed in *Saccharomyces cerevisiae*. However, in the present report we used recombinant cathepsin L1 expressed in the yeast *Pichia pastoris* and purified to high homogeneity as previously reported [18]. Most importantly, this recombinant contained a single amino acid substitution, replacing the active site Cys25 with Gly, with a consequential ablation of functional activity without altering conformation [26] which made the enzyme much more stable in the fermentation and downstream isolation process. Furthermore, because active cathepsin L1 can cleave antibody molecules [6], [25], [27] this modification ensured that the enzyme did not degrade primary and secondary antibodies used in the ELISA.

In our present study we have also optimized the ELISA assay to increase accuracy and reliability. Previous ELISA assays were performed using biotin- conjugated anti-human IgG to detect bound primary antibody followed by anti-human immunoglobulin conjugated with avidin before the substrate ABTS (2,2′-Azinobis [3-ethylbenzothiazoline-6-sulfonic acid]-diammonium salt) was added [18]. The availability of new reagents (HRP-conjugated secondary antibody) have improved the sensitivity of the test, and at the same time decreased the number of steps required and, thus, the work-load and expense.

Optimization of the ELISA was performed by using a pool of sera from patients infected with *F. hepatica* (diagnosed by eggs in stool) and a pool of negative sera from matched non-infected patients. The optimization of the ELISA allowed us to determine the best dilution of the primary sera and secondary antibody to obtain an excellent discrimination between positives and negatives using secondary antibodies that detected total IgG and IgG4. Either of these reagents could be used to diagnose individuals, as proven in previous studies [17], [18], a conclusion supported by the correlation between total IgG and IgG4 depicted in scattergraphs (Figure 4A). Anti-IgG4 exhibited lower background (very low absorbance in Fasciola negative individuals) compared with anti-total IgG; however, statistical analysis illustrated a broader gap between seropositives and seronegatives when using anti-total IgG secondary antibodies which ensures less probability of false-positives or false-negatives.

When using anti-IgG4 as the secondary antibody, two patients (seronegative by anti-total IgG and egg count) appeared as borderline cases; these did not group to the normal seronegative distribution and were beyond the cut-off. In an attempt to include them in the seronegative group the cut off was set at 0.1 OD units and consequently we obtained a narrower gap between positive and negative groups compared to anti-total IgG.

Previous studies by O'Neill *et al.* [18] had shown that by employing anti-IgG4 secondary antibodies led to an improved discrimination between seropositives and seronegatives compared with anti-total IgG. Our contrary results may be explained by the fact that helminthic infections increase anti-IgG4 antibodies in correlation with intensity [27]–[30] and these individuals in the borderline might be at an initial stage of infection or the burden of parasite is low; however, this cannot be ascertained because we do not possess the precise infection levels of the Fasciola-infected individuals from our panel (only presence or absence of eggs were determined). Furthermore, the samples used by O'Neill et al. [18] were obtained from the field and analyzed by anti-IgG4 ELISA using native cathepsin L1. This was a blind study using volunteers in Bolivia which undoubtedly harboured different intensities of infection where the cut-off is more difficult to determine. In the present study we used sera from who had been clinically diagnosed fascioliasis and would therefore have had a high level of infection, and long term infection. This clear distinction between Fasciola positive/negative allowed us to more robustly calculate a cut-off line. It is also possible that different results can be obtained depending on the population of subjects examined. Nevertheless, both the present study and that of O'Neill et al [18] shows that using anti-total IgG provides sufficiently accurate results to consider it the most optimal secondary antibody to use.

We also analyzed the data derived from ELISAs that employed secondary antibodies specific to IgG1 and IgG2 isotypes. However, these secondary reagents did not perform satisfactorily and several patients were misdiagnosed. An increase in the background was observed when using anti-IgG1 and not all Fasciola-infected patients produce IgG1 antibodies. This resulted in an overlapping of some Fasciola-negative and Fasciola-positive sera decreasing the sensitivity and specificity of the test considerably. This was not surprising due to the fact that Fasciola infection induces the production of IgG4 followed by IgG1 and to a lesser extent IgG2 and IgG3 [27].

Sera sample from patients infected with other diseases were used to evaluate cross-reactivity in our ELISA. Analysis of cross-reactivity is extremely important since fascioliasis is a worldwide parasitic disease which can co-exist with other human parasitic diseases which can complicate diagnosis. Furthermore, current parasitological methods depend on the expertise of the worker because *F. hepatica* eggs can be confused with eggs from other helminths. Therefore, a good diagnostic test needs to be able to distinguish between Fasciola and other parasitic diseases. We screened human samples infected with different parasitic diseases and analyzed the ELISA data statistically. Using our ELISAs no cross-reactivity with other parasitic diseases was observed; in fact, the mean absorbances observed for the various diseases examined were not significantly different from the non-infected negative controls patients regardless of whether we employed anti-total IgG or anti-IgG4 secondary antibodies. Moreover, all the Fasciola-infected individuals had significantly higher absorbance readings than those obtained from patients infected with the other parasites. This is consistent with previous studies using native cathepsin L1 [18]. However, we found that anti-total IgG secondary antibody performed slightly better than anti-IgG4 as judged by the gap size between fasciola-positives and fasciola-negatives when the cut off was set.

Over the last two decades there has been a renewed interest in human fascioliasis. This is due to the increase in epidemiological surveys that has revealed the present emergence/re-emergence of the disease both in humans and animals in many regions [26]. Studies have shown that human fascioliasis presents marked heterogeneity, including different epidemiological situations and transmission patterns in different endemic areas [1]. The negative impact of fascioliasis on human communities demands rapid action [2]. Sensitive and specific diagnostic tools are necessary in order to determine the full extent of infections is regions such as Iran, South America and Egypt where animal and human fascioliasis are endemic so that patients can be treated before clinical complications appear.

Here, we have produced a standardized test using a highly stable recombinant form of cathepsin L1, FhCL1, which exhibits high sensitivity and specificity and with no cross-reaction with other parasitic diseases. High production of this enzyme can be obtained by purification of *P. pastoris* culture medium allowing us to provide sufficient quantities of material to supply diagnostic centers for mass screening in regions where human fascioliasis is prevalent.
